# Stress and coping strategies for parenting children with hearing impairment and autism

**DOI:** 10.12669/pjms.36.3.1766

**Published:** 2020

**Authors:** Naima Ishtiaq, Nazia Mumtaz, Ghulam Saqulain

**Affiliations:** 1 Ms. Naima Ishtaiq, M.Phil. (Speech Language Pathology), Speech Language Pathologist, Inspire Education, The Bridge School, Islamabad, Pakistan; 2 Dr. Nazia Mumtaz, PhD. (Rehabilitation Sciences), Head of Department, Department of Speech Language Pathology & Hearing Sciences. Isra Institute of Rehabilitation Sciences, Isra University, Islamabad, Pakistan; 3 Dr. Ghulam Saqulain, F.C.P.S. (Otorhinolaryngology), Head of Department, Department of ENT, Capital Hospital, Islamabad, Pakistan

**Keywords:** Autistic disorder, Copping strategies inventory, Hearing loss, Parental stress scale

## Abstract

**Objectives:**

To determine the level of stress experienced and coping strategies used by parents of hearing impaired and autistic children.

**Methods:**

Using non-probability convenience sampling this cross sectional study recruited n =200 parents of hearing impaired (HI) and 100 parents of autistic children, of either gender, aged 20 to 60 years. Samples were recruited from Special Education Institutes of Islamabad and Rawalpindi, over a period of six months, from October 2018 to March 2019 and conducted at Isra Institute of Rehabilitation Sciences, Islamabad. Basic demographical sheet, Parental Stress Scale and Coping Strategies Inventory were used for data collection. Statistical analysis was done using SPSS 21.

**Results:**

In parents of hearing impaired the mean parental stress score was 47.44±12.85 and commonest coping strategy was problem focused engagement (26.03) followed by problem focused dis-engagement (24.25). In the autistic group the mean parental stress score was 48.92+11.22 with problem focused engagement being the most frequently used strategy (27.4) followed by emotion focused strategy.

**Conclusion:**

Different level of stress experienced by parents of autistic and hearing impaired children which is statistically significant and they employed different coping strategies.

## INTRODUCTION

Stress is part and parcel of any disability,^1^ with lot of stress experienced by parents of children with Autism Spectrum Disorder (ASD)^2^ and hearing impairment (HI).^3^ ASD and HI are common disabilities with the prevalence of HI being 0.1% in newborns,^4^ 1.4% at 5-14 years, 9.8% and 12.2% above 15 years, in males and females respectively.^5^ Hence, HI is the most commonly encountered invisible disability. Compounded with absence of neonatal hearing screening, Pakistan faces late detection and intervention of HI causing parental stress.^6^ Furthermore, this region abounds in joint family system, which can alter the level of stress and coping strategies as well.^7^

Autism is persistent deficit firstly in social communication and interaction including social and emotional reciprocity, nonverbal communication and creating & maintaining relationships; and second in abnormal repetitive behavior, interests and activities including stereotyped speech and behavior, resistance to change, fixated interests and hyper or hyposensitivity to sensory input.^8^ Hearing impairment (HI) may accompany ASD.^9^ Parents live in a heightened state of stress due to additional responsibilities of care of their disabled children in daily living. It is critical to study parental stress in such situations because the increased stress level may affect their health, relation and interactions.^10^ Stress as scientific term defined as a threatened state of homoeostasis due to stressors, counteracted physiological and behavioral responses trying to re-establish the equilibrium. Also the concept of the transactional theory of stress and coping (TTSC),^11^ presents stress as a product of a transaction between a person and environment. Coping Strategies (CS) are basically classified in to three types: Appraisal focused in which persons modifies the way they think; Problem focused eliminating stress by dealing with the cause; and Emotional focused in which emotions that accompany stress are modified.

In the last three decades’ research has focused on stress factors. Researches now focus on the way families cope with these stresses through different strategies. These researches are important in improving, helping and opening new avenues with the care of disabled children.^12^

The present study is imperative due to dearth of local literature and data regarding the prevalence as well as the magnitude of the problem. It could act as a solid base for future research.

## METHODS

This cross sectional study was conducted at Isra Institute of Rehabilitation Sciences, Islamabad after permission of Institutional Review Board (Ref. No. 1609-MPhil SLP-001, dated October 23, 2018). Parental Stress Scale (PSS)^13^ and short version of Coping Strategies Inventory (CSI)^14^ was used with the objective to determine the level of stress and coping strategies used by parents of HI and autistic children. This study included a sample population of n= 200 parents of diagnosed cases HI and n=100 parents of autistic children. Participants were recruited using non-probability convenience sampling from October 2018 to March 2019. Data from parents of HI was collected from persons with special needs school, Dewa Institute of Special & Inclusive Education, National Special Education center for HI, Islamabad; and Sir Syed School and College of Special Education Rawalpindi. While the data of autistic children was collected from Autism Resource Center, Rawalpindi; Rehabilitation center for children with developmental disorders, Inspire child development center, Islamabad. Parents of children with co-morbid conditions or multiple disabilities were excluded. Data was gathered using basic demographical sheet, Parental Stress Scale (PSS) and short version of Coping Strategies Inventory (CSI). PSS is a scale for self-reporting of stress and consists of 18 items each rated on a five-point scale and scored 1 for strongly disagree, 2 for disagree, 3 for undecided, 4 for agree, 5 for strongly agree. The possible score on PSS is between 18 -90 with higher scores indicating higher stress. CSI consists of 32 items represented on three levels. Following consent for inclusion in the study, the research instrument was self-administered by the researcher.

Data was entered in excel worksheet and coded and statistical analysis done by SPSS Version 21. Descriptive analysis was done by using frequency distribution and percentage. Mean and standard deviation was used to elaborate the results of PSS and CSI.

## RESULTS

The study population comprised of 31.33% males and 68.67% females and mean age of 40.56±6.87 years. Study revealed a high parental stress with a mean score of 47.44±12.85 in HI group and 48.92±11.22 in the Autistic group (Table-I). In parents of HI problem focused engagement was commonest coping strategy (26.03%) followed by problem focused dis-engagement (24.25%), While in case of autistic children, problem focused engagement was commonest strategy (27.4%) followed by emotion focused strategy (23.95%).

**Table-I:** Variables of Parents of HI and autistic children *Parental Stress Scale (PSS) & Coping Strategies Inventory (CSI) Score (Mean & SD). Cross Tabulation. (N=300).

		Parental Stress Score		Engagement		Dis-Engagement	

Variable		Mean	SD	Problem Focused (N)	Emotion Focused (N)	Problem Focused (N)	Emotion Focused (N)
Disability	HI	47.44	12.85	26.03	22.04	24.25	18.8
	Autism	48.92	11.22	27.4	23.95	22.34	19.63
Number of Special Children	1 (n=181, 60.34%)	41.52	8.16	27.06	23.06	23.86	18.02
	2 (n=72, 24.32%)	49	11.11	25.73	21.41	23.8	18.32
	3 (n=2, 7%)	57.42	6.56	24.15	22.23	28.23	26.85
	4 (n=17, 5.67%)	63.45	5.14	18.75	16.5	31.75	30.25
	5 (n=8, 2.67%)	75.17	7.98	15.5	18.5	34.5	34
Disability Level (HI)	Mild	36.9	6.91	24.22	22.67	18.11	16.56
	Moderate	43.84	6.58	28.14	22.45	23.72	19.38
	Severe	47.2	11.43	21.52	19.81	26.63	23.56
	Profound	60.62	11.96	22.26	21.77	24.5	26
Disability Level (Autistic)	Mild	35.5	8.22	32.12	27.28	20.5	19
	Moderate	46.22	7.14	27.42	24.52	22.31	18.79
	Severe	60.84	6.4	23.83	22.83	26.5	29.67

Parental stress was lowest with one special child and maximum in parents of 5 special children. With 1-2 special kid the coping strategy most commonly used was problem focused engagement followed by problem focused dis-engagement, while with 3-5 special children the most commonly used strategy was problem focused disengagement followed by emotion focused disengagement.

As regards level of stress experienced by fathers of hearing impaired with respect to the educational level, the under graduate had high stress scores as compared to graduate fathers with Mean score of 50.68±14.76 vs. 46.12±17.1). In the autistic group the graduate fathers had a PSS score mean of 51.67±13.58, while there was no parent in the under graduate group. ANOVA test statistics revealed statistically significant difference between autistic and HI group (P <0.001). As far as mothers are concerned, for mothers of HI and autistic group the PSS scores of un-graduate were high as compared to graduate with mean score of 48.37±10.2 vs. 41.82±9.6 for HI and 51.83±12.6 vs. 45.78±10.4 for autistic group with statistically significant correlation between scores of autistic and HI mother (p< 0.001).

Problem focused engagement was the most frequently used coping strategy in parents of both HI and autistic parents compared to emotional focused engagement with mean scores of 26.03 vs. 22.04 for HI and 27.4 vs. 23.95 for autism group (Table-II).

**Table-II:** Breakup of Coping Strategies Inventory (CSI) Score *Gender, Education Level and Disability Type. Cross Tabulation. (N=300).

Dimension		Statistics	Engagement		Dis-Engagement	

			Problem Focused	Emotion Focused	Problem Focused	Emotion Focused
Hearing Impaired			26.03	22.04	24.25	18.8
Male	Under Graduate	Mean	25.23	21.78	23.83	20.21
		SD	7.18	5.75	5.8	8.45
	Graduate	Mean	27.375	24.42	21.67	14.83
		SD	6.55	4.9	3.96	4.18
Female	Under Graduate	Mean	25.54	20.65	25.9	20.2
		SD	6.05	4.47	4.77	8.02
	Graduate	Mean	27.44	23.41	23.76	16.5
		SD	5.82	6.34	5.6	7.24
Autism			27.4	23.95	22.34	19.63
Male	Under Graduate	-		-	-	-
	Graduate	Mean	28.67	24.16	23.83	19.83
		SD	5	1.72	3.18	4.07
Female	Under Graduate	Mean	21.16	20.16	23.33	25.33
		SD	2.83	3.31	3.83	2.06
	Graduate	Mean	28.34	24.68	21.81	18.53
		SD	5.45	3.6	6.74	6.47

It is observed that scores of problem focused engagement of graduate mother was significantly higher than those of under graduate mothers. A significant finding is that problem focused dis-engagement was most commonly use strategy with under graduate HI mothers with a mean score of 25.90±4.77, while in the autism group dis-engagement strategy was most commonly used by under graduate mothers with emotion focused disengagement used more frequently (25.33±4.07) then problem focused dis-engagement (23.33±3.83). Problem focused engagement was the most frequently used strategy in father of HI with significantly high score of problem focused engagement in graduate fathers with mean score of 27.37 & 6.55. While problem focused disengagement was the frequently used in under graduate fathers of with mean score of 23.83 & 5.80.

The third level of coping strategies inventory revealed difference between dimensions of coping strategies with higher mean scores of engagement strategies compared to dis-engagement (47.55±9.93 vs. 43.3±11.43) for the HI group. Similarly, in the autism group, higher mean scores of engagement strategy were noted compared to dis-engagement strategy (50.47±6.66 vs. 41.97±9.27).

To verify whether the use of these strategies would differ according to education and type of disability, means and standard deviations of Educational scores on coping strategies inventory was used in two-way ANOVA analysis to investigate the variance between these mean. Results of this analysis, revealed statistically significant difference with p < 0.0001.

## DISCUSSION

Birth of a special child is a distressing event having emotional, physical and social impact on the caregivers,^15^ with higher level of stress in parents of children with disability.^16^ Current study revealed a high parental stress level with a mean score of 47.44 in HI group & 48.92 in the autistic group. A high levels of stress in parents of disabled with less stress level in parents of HI compared to physical disabilities has been reported by Bawalshah.^17^

In current study, as regards level of stress experienced by fathers of hearing impaired with respect to the educational level, the under graduates had high stress scores as compared to graduate fathers. While in mothers of HI the PSS scores of un-graduate were high as compared to graduate. Similarly, in a study by Vinayak et al. mothers of HI were found to be more stressed.^18^ In contrast another study reported that mothers did not feel high level of general parenting stress, except for HI.^19^ In present study, in the autistic group the graduate fathers had a PSS score mean of 51.67, while there was no parent in the under graduate group. A high PSS score was noted for undergraduate mothers of autistic children compared to graduates. While in study by Dervishaliaj et al., a gender differences were observed with more stress suffered by mothers.^10^

Prata J et al. reported high stress levels in ASD children related to factors including characteristics of parents, children, support of family, and professional, economic and social support.^20^ Similarly in a local study moderate level of stress was reported in parents of HI compared to intellectually challenged where profound stress was reported.^21^ Ebrahimi H et al. reported that parents of HI live with fear of having another HI baby in the next pregnancy.^22^ In the present study the number of special children seem to be related to increase in parental stress with minimum stress with one special child. With one to two special children the coping strategy most commonly used included problem focused engagement, while with 3-5 special children the most commonly used strategy was problem focused disengagement. Gupta VB et al.^23^ reported that female special child causes more parental-child dysfunction. Also parents on prestigious, lucrative jobs had more stress. Gupta found a striking finding contradicting previous notion, that extended families in India, supposed to be closely knit and considered largely supportive, were not like this on real grounds and after having negative experiences from different corners strata of society and exhausting the treatment modalities, majority turn towards God, mosques and temples as a copping strategy and see disability as the will of the God.^23^ In a study by Jenaabadi, targeting parents of visual, hearing and mentally impaired children, revealed high mean stress score with equal use of coping strategies.^24^

We also found that problem focused engagement was the most frequently used compared to emotional focused engagement. Problem focused engagement was the most frequently used strategies in both mothers of HI and autistic children with scores of graduate mother being significantly higher than those of under graduate mothers. A significant finding is that problem focused dis-engagement was most commonly use strategy with under graduate HI mothers, while this was not so in the autism group where dis-engagement strategy was most commonly used by undergraduate mothers with emotion focused disengagement used more frequently then problem focused dis-engagement. Cappe E et al. in their study on ASD found negative impact of emotion-focused coping strategies, with parents becoming more stressed and disturbed and highlighted the need of psycho-education, modifying false beliefs and focused management of stress and emotion of parents.^25^

In current study, problem focused engagement was the commonest strategy in father of HI with significantly high score of problem focused engagement in graduate fathers. While problem focused disengagement was the frequently used in under graduate fathers. In a study by Bawalsah JA, reported that engagement coping strategies were more commonly used in parents, with focus to use problem focused engagement more than emotion focused engagement strategies, while fathers favored using engagement strategies compared to mothers who favored disengagement strategies.^17^

Mostafa MH, reported moderate stress in 60% of parents of autistic children and 50% of these were using coping strategies including seeking information, avoidance and denial and a significant correlation between level of stress and coping strategies was also noted.^26^ Stoia D et al. studied the usefulness of Emotionally Focused Therapy (EFT) and reported studies that questioned its effectiveness for ASD.^27^ Turns B et al. reported effectiveness of solution-focused brief therapy for coupes of autistic children and improves well-being and marital satisfaction.^28^

Ntre V et al.^29^ in a Greek study reported that mothers of children with ASD have psychological and financial issues, with the spouse being the main support. Neyoshi et al. in a study concluded that support tailored to local characteristics and values of parents can build support systems that are acceptable to parents.^30^ A Chinese study for parents of autism the results indicate benefited from the support group for sharing experiences, sense of belongingness and feeling of relating to one another.^31^ Gupta and Singhal in their study noted that positive reframing and cognitive reframing can lead to positive handling and coping strategies.^32^

## CONCLUSIONS

There exist statistical differences in level of stress experienced by parents of Autistic and Hearing impaired children and they employed different coping strategies to deal with it. This study may be beneficial for professionals working on planning and management of any one of the major disability by intervening at the right time and implementing coping strategies to reduce and manage parental stress.

### Authors’ Contribution:

**NI,** conceived, designed the research and methodology and collection of data.

**NM,** did the data analysis, interpretation and critical revision of the article.

**GS** did the writing of manuscript, literature review and responsible for final publication and takes the responsibility and is accountable for all aspects of the work including the accuracy and integrity of all parts of the work

## References

[1] Khan S (2017). Stress in the parents of children with physical disability. J Pak Psychiatr Soc.

[2] Bonis S (2016). Stress and Parents of Children with Autism:A review of literature. Issues Ment Health Nurs.

[3] Shalini B, Rout D, Srivani G, Sridevi G (2017). Burden and Social Support of the Caregivers of Children with Hearing Impairment. Int Multidiscip E-J.

[4] Ulusoy S, Ugras H, Cingi C, Yilmaz HB, Muluk NB (2014). The results of national newborn hearing screening (NNHS) data of 11,575 newborns from west part of Turkey. Eur Rev Med Pharmacol Sci.

[5] Stevens G, Flaxman S, Brunskill E, Mascarenhas M, Mathers CD, Finucane M (2013). Global Burden of Disease Hearing Loss Expert Group. Global and regional hearing impairment prevalence:an analysis of 42 studies in 29 countries. Eur J Public Health.

[6] Mumtaz N, Habibullah S (2017). Better late than never:Identification of children with hearing loss in Pakistan. Pak Armed Forces Med J.

[7] Ahmed S, Ali J, Sanauddin N (2016). Patriarchy in family care-giving:Experiences of families of children with intellectual disability in Pakistan. J Postgrad Med Inst.

[8] Lee PF, Thomas RE, Lee PA (2015). Approach to autism spectrum disorder:Using the new DSM-V diagnostic criteria and the CanMEDS-FM framework. Can Fam Physician.

[9] Demopoulos C, Lewine JD (2016). Audiometric Profiles in Autism Spectrum Disorders:Does Subclinical Hearing Loss Impact Communication?. Autism Res.

[10] Dervishaliaj E (2013). Parental stress in families of children with disabilities:A literature review. J Educ Soc Res.

[11] Lee E, Roberts LJ (2018). Between individual and family coping:a decade of theory and research on couples coping with health-related stress. J Fam Theory Rev.

[12] Baker JP, Berenbaum H (2007). Emotional approach and problem-focused coping:A comparison of potentially adaptive strategies. Cognition Emotion.

[13] Zelman JJ, Ferro MA (2018). The Parental Stress Scale:Psychometric Properties in Families of Children with Chronic Health Conditions. Fam Relat.

[14] Speyer E, Morgenstern H, Hayashino Y, Kerr PG, Rayner H, Robinson BM (2016). Reliability and validity of the coping strategy inventory-short form applied to hemodialysis patients in 13 countries:Results from the Dialysis Outcomes and Practice Patterns Study (DOPPS). J Psychosom Res.

[15] Gallagher S, Phillips AC, Oliver C, Carroll D (2008). Predictors of psychological morbidity in parents of children with intellectual disabilities. J Pediatr Psychol.

[16] Nadeem M, Choudhary FR, Parveen A, Javaid F (2016). Parental stress among parents of children with and without disabilities. Pak J Soc Sci.

[17] Bawalsah JA (2016). Stress and Coping Strategies in Parents of Children with Physical, Mental, and Hearing Disabilities in Jordan. Int J Educ.

[18] Vinayak S, Dhanoa K, Vinayak R (2016). Relationship of hopelessness, depression and quality of life in mothers of persons with disabilities. Int J Innov Appl Stud.

[19] Lederberg AR, Golbach T (2002). Parenting stress and social support in hearing mothers of deaf and hearing children:A longitudinal study. J Deaf Stud Deaf Educ.

[20] Prata J, Lawson W, Coelho R (2019). Stress factors in parents of children on the autism spectrum:an integrative model approach. Int J Clin Neurosci Ment Health.

[21] Firdous N, Mumtaz N, Saqulain G (2019). Psychological stress among parents of hearing impaired versus intellectually disabled Pakistani children. J Islamabad Med Dental Coll.

[22] Ebrahimi H, Mohammadi E, Pirzadeh A, Shamshiri M, Mohammadi MA (2017). Living with Worry:The Experience of Mothers with Deaf Child. Int J Pediatr.

[23] Gupta VB, Mehrotra P, Mehrotra N (2012). Parental stress in raising a child with disabilities in India. Disability, CBR &Inclusive Development.

[24] Jenaabadi H (2014). The Study and Comparison of Stress Levels and Coping Strategies in Parents of Exceptional (Mentally Retarded, Blind and Deaf) and Normal Children in Zahedan. Procedia Soc Behav Sci.

[25] Cappe E, Wolff M, Bobet R, Adrien JL (2011). Quality of life:A key variable to consider in the evaluation of adjustment in parents of children with autism spectrum disorders and in the development of relevant support and assistance programmes. Qual Life Res.

[26] Mostafa MH (2019). Stress and Coping Strategies among Parents of Children with Autism Spectrum Disorder. PEOPLE:Int J Soc Sci.

[27] Stoia D, Vintila M, Minulescu M (2019). Couples Facing the Autism Spectrum Disorder Challenge:A literature review concerning emotionally focused therapy effectiveness. Soc Res Rep.

[28] Turns B, Jordan SS, Callahan K, Whiting J, Springer NP (2019). Assessing the Effectiveness of Solution-Focused Brief Therapy for Couples Raising a Child with Autism:A Pilot Clinical Outcome Study. J Couple Relationship Ther.

[29] Ntre V, Papanikolaou K, Triantafyllou K, Giannkopoulos G, Kokkosi M, Kolaitis G (2018). Psychosocial and Financial Needs, Burdens and Support, and Major Concerns among Greek Families with Children with Autism Spectrum Disorder (ASD). Int J Caring Sci.

[30] Neyoshi C (2018). Public Health Nurses'Support for Children with Autism Spectrum Disorder (ASD) and their Parents, Tailored to the Level of Parental Acceptance and Local Characteristics. J Comm Pub Health Nurs.

[31] McCabe H, McCabe K (2013). Disability and family in the People's Republic of China:Implementation, benefits, and comparison of two mutual support groups. J Intellect Dev Disabil.

[32] Gupta A, Singhal N (2004). Positive perceptions in parents of children with disabilities. Asia Pacific Disabil Rehabil J.

